# Integrating systems thinking for analyzing and designing national early warning surveillance for animal health: a perspective from Tanzania

**DOI:** 10.3389/fvets.2026.1787091

**Published:** 2026-07-22

**Authors:** Janeth George

**Affiliations:** Department of Economics and Community Economic Development, Faculty of Arts and Social Sciences, The Open University of Tanzania, Dar Es Salaam, Tanzania

**Keywords:** animal health surveillance, early disease detection, One Health, socio-technical systems, veterinary epidemiology

## Abstract

Animal health surveillance has demonstrated its value in global health security. The effectiveness of a surveillance system in reducing health threats and their impact depends on complex interactions between processes, enablers and context. However, most national animal health surveillance systems, including those in Tanzania, still face complex, interconnected challenges that undermine their efficiency. This paper is presented as a conceptual perspective that synthesizes insights from previously published studies on the use of integrative approaches for analyzing, designing, and evaluating animal health surveillance systems. The studies focused on evaluating the system, identifying data sources, mapping stakeholders for collaboration, and developing a prototype of an integrated animal health surveillance system. The paper offers a structured socio-technical framework drawing on experience from Tanzania, a resource-limited setting. It highlights the complex interplay between technical and social factors that shape the implementation of animal health surveillance. Strengthening surveillance systems required the adoption of technologies that facilitate integration and interoperability of multiple data sources for continuous improvement on system attributes, decision outcomes and ultimately health outcomes. It is also critical to consider social aspects, such as investment in human and financial resources, and foster collaboration between public and private stakeholders to improve data flow from various sources. Most importantly, there should be capacity building in using data for decision-making to enhance a timely response to health threats. This paper provides additional guidance to countries in assessing their current surveillance setups and developing more efficient early warning systems.

## 1 Introduction

### 1.1 Background

In the animal health context, surveillance is defined as the systematic, continuous or repeated measurement, collection, collation, analysis, interpretation and timely dissemination of animal health, and welfare-related data from defined populations (1). Surveillance helps decision-makers to manage disease prevention and control more effectively by providing timely and useful evidence for targeted action (2). In recent years, there has been a growing concern about the spread of infectious animal diseases, as they impact animal welfare, threaten public health, and affect economies at both micro and macro levels. Therefore, effective surveillance enables early detection, prevention and control of animal diseases, including zoonoses (3, 4).

Animal health surveillance has demonstrated its value in global health security, including predicting public health risks, emphasizing the importance of having stronger systems at national, regional, and global levels. It can provide early warnings of emerging threats to human populations from bioterrorism or naturally occurring infectious disease epidemics (5, 6) through sentinel surveillance, mapping of the spatial-temporal distribution of animal hosts and survey of animal reservoirs (7). Animal health surveillance has also been used to address food safety issues and control antimicrobial resistance challenges (8).

However, surveillance is an ever-evolving activity that needs to be backed up by the best available scientific evidence and methodology (9). Novel approaches have been developed to enhance surveillance systems at national and international levels for early detection and response to disease outbreaks. This includes exploring participatory and syndromic surveillance (10–13) and integrating data across the systems (14, 15). Most surveillance systems have moved beyond focusing solely on infectious diseases to include monitoring and predicting a wide range of health determinants (16). However, the literature indicates that innovations in animal health surveillance are new and few compared to public health (7, 17) and they still face challenges.

The effectiveness of a surveillance system in reducing health threats and their impact depends on complex interactions between processes, enablers, and context. Therefore, when evaluating the performance of the system, it is important to focus beyond the surveillance processes into the contextual and structural factors and their relationship to the system. To do that, one must use systems thinking approaches which focus on understanding why a system works that way (system synthesis) rather than how it works (system analysis) (18). Systems thinking theories propose an understanding of a system by holistically examining the linkages and interactions between the elements that compose the entire system (19). In the context of animal health, surveillance is regarded as a complex adaptive system with dynamic interactions among its components and between these components and their external environment. Therefore, understanding it in a systems lens helps to identify critical and leverage points for improving performance.

Tanzania is among the African countries with the highest number of livestock and a large wildlife population, with great interaction among them, especially around national parks and game reserves. This makes it vulnerable to animal disease outbreaks, including zoonoses. Since independence in 1961, the country has made significant strides in animal health surveillance, including the establishment of a nationwide Veterinary laboratory network in 2012, the training of veterinarians, livestock field officers, and laboratory technicians, and attempts to migrate to digital technology-enabled surveillance (20). There are also policy and legal frameworks that guide animal health surveillance, including the Animal Disease Act (2003), Veterinary Act 16 (2003), Livestock Identification, Registration and Traceability Act (2010), Livestock Policy (2006), Animal Health Surveillance Strategy (2019–2023) and National Strategic Plan for One Health (2015–2020). However, several evaluations conducted between 2008 and 2017 including Performance of Veterinary Services (21), Joint External Evaluation (22), FAO surveillance evaluation tool (23) and national audit report (24) indicated that the national animal health surveillance system still had a limited capacity to detect and respond to disease outbreaks due to complex interconnected challenges.

Systems thinking provides a framework for examining the factors involved in a problem, the relationships between these factors, and changes over time; it views actions as integrated across political, social, cultural, economic, and scientific domains within a system (25). Therefore, a series of studies guided by socio-technical systems theory were conducted between 2017 and 2021 to explore how the animal health surveillance system in Tanzania could be improved through integrative approaches that embrace systems thinking. This paper is presented as a conceptual perspective that synthesizes insights from previously published studies (26–29) on the use of integrative approaches for analyzing, designing, and evaluating animal health surveillance systems. The studies focused on evaluating the system, identifying data sources, mapping stakeholders for collaboration, and developing a prototype of an integrated animal health surveillance system. This paper offers a structured socio-technical framework drawing on experience from Tanzania, a resource-limited setting.

### 1.2 Application of socio-technical systems theory in the context of animal health surveillance

Underpinned by systems thinking, socio-technical systems theory (STS) embraces the idea that all aspects of the systems are interconnected, that none should take logical precedence over the other, and that they should be designed jointly (30). It explores the relationship between components of a complex system as a whole (31). In recent years, there has been a growing interest in exploring the integration of socio-technical theory into system design (32–34), acknowledging the limitations of the reductionist and siloed approaches to systems analysis and design. There is also increasing evidence on how interaction between technical components and people has improved system performance, such as enhanced creativity (35), improved digital transformation (36) and developed trust among users (37).

**Figure 1:**
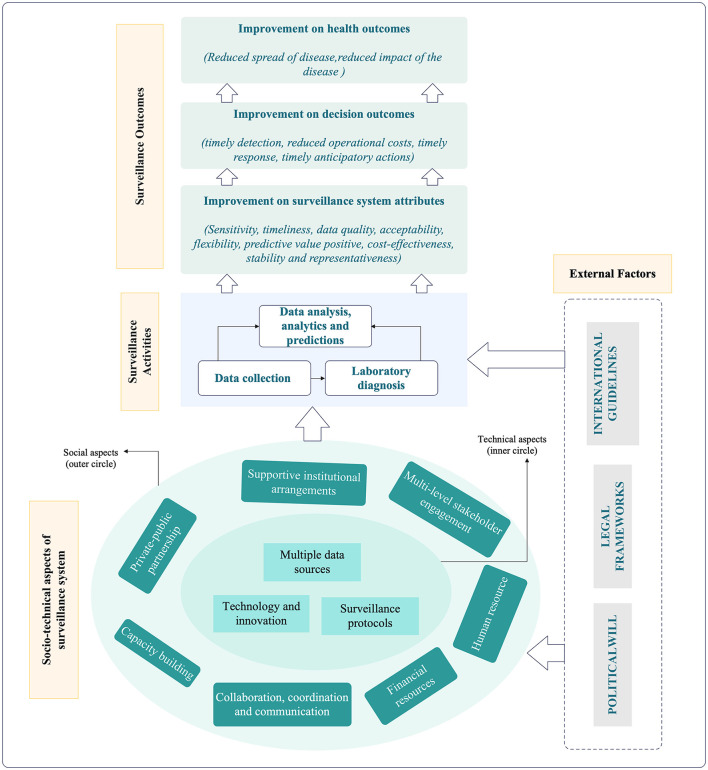
The animal health surveillance system is a complex adaptive system. The system is made up of three agents (socio-technical aspects to consider [inner and outer green circles], surveillance activities [blue box] and outcomes [pale mint boxes]) which are interrelated and interact to each other and its external environment (political will, legal frameworks, and international guidelines) ultimately lead to achievement of the overall impact (reduced impact of the disease).

In the context of animal health, a surveillance system is regarded as an open system characterized by interaction between components and the environment (32). Furthermore, an animal health surveillance system can be conceptualized into three subsystems: social, technical and environmental (32). In this case, the social subsystem encompasses human factors such as institutional arrangements, stakeholder relationships and interactions, communication, coordination, collaboration, and capacity building. Technical subsystems include data sources, data collection tools and processes, data management and analysis, laboratory diagnostics, surveillance protocols and guidelines, technology and innovations, human resource capacity, and financial resources. However, the system is influenced by complex environmental factors such as psychological, economic, technical, cultural, and political which need to be understood (38). In the study, the animal health surveillance system was further made up of three main agents: inputs, enablers and outcomes, which are interrelated and interact with each other and their external environment (Figure 1)

It should be noted that open systems acknowledge that interaction among and within components, as subsystems, and with their environment is constant and flexible. Therefore, in the context of animal health surveillance, boundaries are defined by the objective of the system, institutional mandates and system actors.

## 2 Technical aspects to consider in designing national early warning surveillance for animal health

### 2.1 Integrative approaches for analyzing animal health surveillance systems

A process evaluation study, which assessed processes, mechanisms and contextual factors affecting implementation of animal health surveillance in Tanzania (28), revealed that there is a complex interaction between mechanisms and contextual factors that collectively or individually influence system processes and outcomes. For instance, the participation level of livestock field officers in surveillance activities (mechanism) and the coverage of designated areas (context) influence adherence to the reporting timeline (process), which ultimately affects data timeliness (outcome). To understand the complexity of these interactions within a system like animal health surveillance, it is essential to adopt a systems perspective, which is one of the integrative problem-solving approaches. Progress has been made in using integrative methods to assess animal health surveillance systems, but there are still few tools available to guide them (39). To illustrate the use of systems thinking, the study adapted the process evaluation framework by Moore et al. (40) originally designed for examining complex health interventions but not previously applied to surveillance systems.

### 2.2 Looking beyond disease surveillance by leveraging multiple data sources

Most surveillance systems were designed to detect diseases, making it difficult to identify abnormal patterns that don't fit standard case definitions. This approach is resource-intensive and requires prior knowledge of disease likelihood (41). Furthermore, focusing only on known pathogens could lead to overlooking emerging health threats that might potentially become pandemics. As the world moves toward early warning systems, it is also vital to capture drivers and signals that could alert decision-makers to take early action. Using predefined criteria, a mixed-methods study was conducted to identify and assess existing and potential data sources for integration into an animal health surveillance system (26). Thirteen data sources were identified and assessed against six criteria: data content, spatial coverage, frequency of collection, accuracy, cost, and accessibility. The majority of the data sources had minimum to maximum scores for content, spatial coverage, and accessibility, but there were significant variations in frequency, accuracy, and cost. The common variables found across most sources were: the name of the place (12/13), animal type/species (12/13), syndromes (10/13) and number of affected animals (8/13). This indicates that each data source may have strength in some aspects, such as timeliness, but weakness in others, for instance, specificity. Therefore, leveraging multiple data sources has potential in strengthening animal health surveillance through trade-offs between strengths and weaknesses.

### 2.3 Strengthening early warning capabilities of animal health surveillance systems through interoperability

Timely information about health events is a fundamental aspect of an early warning surveillance system, aiding informed decision-making and prompt action. A systematic review found that interoperability is the most preferred system integration mechanism (29). It enables the system or its components to work with other systems without much effort from users, making it more convenient and safer, as it doesn't require merging the systems but rather synchronization. For instance, an animal health surveillance system may be interoperable with meteorological information systems to predict climate-linked vector-borne diseases using only an Application Programming Interface (API).Taking into account the strength of interoperability and contextual factors, the prototype of an interoperable system named *W*anyama he*A*lth su*R*veilla*N*ce (WARN) was developed using Hypertext Preprocessor (PHP) version 7.4 (Laravel framework), Python version 3.8.0 and MySQL database (42). It gathers and integrates laboratory data captured in *Sistema Informativo di Laboratorio-*Laboratory Information Management System (SILAB-LIMS), case-based event reports through the Event Mobile Application (EMA-i)/EMPRES-I, and data from field operations and syndromic data from the community and private veterinary facilities, captured through AfyaData. Three animal health information systems in Tanzania were linked through Application Programming Interfaces (APIs). The practical application of the prototype was demonstrated by simulating it with sample records and dummy data pulled from integrated systems and visualizing its output on the unified interactive dashboard. The prototype was adapted by Tanzania's Ministry of Livestock and Fisheries, with support from the Food and Agriculture Organization of the United Nations (FAO) and technical support from SACIDS Foundation for One Health, to develop integrated animal health surveillance systems and was renamed UTAMBUZI.

### 2.4 Designing an animal health surveillance system in the framework of One Health

It is now widely recognized that 60% of human diseases are zoonotic, transmitted from animals (43). Developing animal health surveillance within the One Health framework is a crucial step toward tackling diseases at their origin. The One Health approach emphasizes the interconnections between human, animal, and environmental health, providing a platform for stakeholders from diverse sectors, disciplines, and communities to collaborate on health and ecosystem challenges (44). In one of the studies, stakeholder mapping was conducted to explore the existing collaboration and influences at subnational levels that can be leveraged to improve the national animal health surveillance system (27). Data were collected using an adapted USAID stakeholder collaboration mapping tool (45) and stakeholders were grouped into four categories: private sector and non-government organizations (NGOs), government, community and political leaders mapped against resource and non-resource-based influence. Through this study, it was demonstrated that the private sector and NGOs have stronger resource-based influence while political leaders have the strongest non-resource-based influence. Therefore, to mobilize communities to participate in surveillance and one health challenges and ensure seamless data flow, it is important to consider all categories of stakeholders.

The world is moving toward semantic and convergent integrations that require high-level standardization of data. However, a systematic review revealed that animal health remains slow in achieving intersectoral systems integration (29). An effective integrated system enables prevention, early detection, and prompt response to health threats, thereby reducing their impact. The WARN prototype demonstrated that by developing a flexible, interoperable system capable of integrating large datasets, it contributes to the advancement of One Health surveillance and intelligence systems. These systems collect data on human, animal, and environmental health to enhance global health security.

## 3 Social aspects to consider in operationalizing national early warning surveillance for animal health

### 3.1 Capacity building on surveillance data use for decision making

In recent years, there has been significant progress in integrating surveillance systems, but this work has mostly focused on technical aspects, including data collection, management, and analysis. Yet, interpretation and use of those data to make informed decisions is another critical component but gets less attention. The systematic review conducted revealed that integration comes with large data volumes that require the capacity to package and disseminate surveillance information generated from the systems to decision makers for them to take appropriate action. A process evaluation of Tanzania's animal health surveillance system found that, despite livestock field officers spending up to 90% of their time on surveillance activities, they rarely participate beyond data collection and seldom receive training. Surveillance data were mainly used at the national level for designing control and prevention programs (28). This may in a way contribute to the low performance of the systems especially on the data quality and reporting rate. Therefore, as we are moving toward technology-enabled integration, it is important to create a pool of workforce who understand surveillance processes from data collection to intervention/action.

### 3.2 Financial investment in animal health surveillance systems

Animal health surveillance systems require financial resources to support the development of tools, data collection processes and system setup. However, in most low-income countries, animal health surveillance budgets are heavily dependent on public and donor funding. In Tanzania, the livestock sector is regarded as a productive sector; therefore, the Directorate of Veterinary Services doesn't receive a sufficient budget compared to the budget allocated to human health. The government's annual budget for surveillance is less than 5% of the total ministerial budget, which greatly impacts surveillance efforts (28). To reverse this trend, conducting economic evaluations of surveillance systems is necessary to advocate for more investment.

### 3.3 Fostering public-private partnership in surveillance

In Tanzania, animal health is largely a private practice overseen by the Veterinary Council of Tanzania (VCT). A significant amount of information is generated through routine practices; however, it has not been mainstreamed into surveillance. They only report notifiable diseases as required by the Animal Disease Act of 2003. There are a number of agrovet shops registered that could be leveraged. The study showed that before dispensing veterinary drugs, the shop attendant asks some basic questions that can help capture early signals of what is circulating on the ground (26). Therefore, by strengthening public-private partnerships, private practitioners can contribute to surveillance data collected through their routine activities, thereby reducing the transactional cost of data. Furthermore, stakeholder mapping suggested that the private sector could contribute to funding surveillance activities through in-kind contributions of their resources (27).

## 4 Discussion

Current surveillance models, especially in animal health, remain fragmented with weak to no integration. Most surveillance systems are highly reliant on passive surveillance, which leads to underreporting, delayed threat detection, and slower responses. This study demonstrated the value of systems thinking approaches in analyzing and designing proactive and adaptive animal health surveillance systems that would effectively provide early warning, particularly within the One Health framework.

Systems thinking approaches have proven valuable in providing a broader perspective for identifying and solving complex problems such as food insecurity (46), community health (47) and effect of climate change in livestock farming (48). However, little has been done in animal health surveillance. There are no clear guidelines or tools for implementing it. This study suggests that using socio-technical systems theory could offer a starting point for exploring how technical and social components individually and interactively influence system performance. While examining the technical aspects of the system, such as data sources and business processes, it is equally important to consider social factors, particularly how people interact with the system, as well as financial resources, partnerships, and capacity building.

When designing an early warning surveillance system, it is crucial to identify leverage points. For instance, using digital technologies can enhance the intelligence capabilities of the system in capturing signals (49, 50). It is equally important to integrate veterinary service structures, such as private practitioners and agrovet shops to maximize data flow. Furthermore, leveraging informal reporting channels can strengthen event and community-based surveillance which are also vital aspects of early warning systems (51, 52). However, all these measures rely on a well-established feedback loop that closes the gap between reporting and response. The most important thing is to ensure that no stakeholder is left behind through periodic mapping and updating of animal health stakeholders in the country.

Despite its benefits, integrating systems thinking into surveillance system analysis and design may be slow due to the current and anticipated challenges (53, 54), especially in low-resource settings. Unpacking complex systems like animal health surveillance requires time and resources, thereby necessitating institutional commitment to investigate and go into the depths of technical and social factors that positively and negatively influence the efficient operationalization of the surveillance systems. On the other hand, complete integration of data and systems will have to be gradual and stepwise while addressing data limitations and enabling people to understand and comfortably interact with the system. Stakeholders may use adaptive management and learning for continuous system improvement.

As we advance system thinking for early warning surveillance for animal health, it should go hand in hand with investment in human and digital technologies. It is important to equip practitioners with practical skills and tools for analyzing complex systems and design appropriate interventions. To match data influx due to integration of data sources of which some of them may be unstructured, they should also be trained on how to leverage artificial intelligence, machine learning and other analytics tools to make sense of the data and present it in a language that can be understood by non-technical decision makers such as politicians.

Integrating systems thinking into animal health surveillance should promote intersectoral and multisectoral collaboration and the institutionalization of systems-based approaches within surveillance frameworks (55). Animal health surveillance stakeholders, particularly researchers, policymakers, and practitioners, should commit to continuously enhancing human resource capacity and improving data infrastructure within the framework of One Health as a decision-making tool for preventing, detecting, and responding effectively to emerging and re-emerging animal and public health threats.

## 5 Conclusion

Increased global health threats to animals and humans necessitate the need for early warning surveillance systems. However, given the overwhelming limitations in most surveillance systems, it is critical to apply systems thinking to unpack such complex systems. Guided by Socio-Technical systems theory, this paper demonstrates the application of systems thinking as one of the integrative approaches for evaluating and designing effective early warning surveillance for animal health and other sectors. It reveals how both social and technical factors interactively affect the system and identified leverage points for enhancing performance. However, it is worth noting that most of the animal health surveillance systems remain unintegrated. Therefore, this perspective paper may guide countries in assessing their current surveillance setups and developing more efficient early warning systems.

## Data Availability

The original contributions presented in the study are included in the article/supplementary material, further inquiries can be directed to the corresponding author.
